# Single-Cell Transcriptomic Analysis of Salivary Epithelial Cells Reveals Large-Scale Dysregulation in Bitter Taste Dysfunction

**DOI:** 10.3390/ijms27072953

**Published:** 2026-03-24

**Authors:** Shveta Jaishankar, David Schaeper, Sarath Chandra Janga, Mythily Srinivasan

**Affiliations:** 1 Department of Oral Pathology, Medicine and Radiology, Indiana University School of Dentistry, Indianapolis, IN 46202, USA; shjaish@iu.edu; 2 Department of Biomedical Engineering and Informatics, Luddy School of Informatics, Computing and Engineering, Indiana University Indianapolis, 535 West Michigan Street, Indianapolis, IN 46202, USA; daschaep@iu.edu (D.S.); scjanga@iu.edu (S.C.J.); 3 Center for Computational Biology and Bioinformatics, Indiana University School of Medicine, 5021 Health Information and Translational Sciences (HITS), 410 West 10th Street, Indianapolis, IN 46202, USA

**Keywords:** scRNA, saliva, epithelial cells, long COVID, taste dysfunction, bitter

## Abstract

Taste dysfunction, or dysgeusia, is a frequent symptom associated with infections and systemic diseases, yet its cellular and molecular basis remains poorly understood. The COVID-19 pandemic provided an opportunity to study dysgeusia as a “natural experiment” due to its high prevalence in those with acute and long COVID (LC). We investigated salivary epithelial cells (SECs) using single-cell RNA sequencing to elucidate molecular changes underlying taste dysfunction in LC. Functional enrichment analysis of SEC transcriptomes from individuals with bitter taste dysfunction (LC-D) revealed downregulation of genes involved in cytoskeletal dynamics and taste cell–nerve synapse assembly. Further, specific Type II and III taste receptor genes, critical for bitter taste perception, were reduced. Microbial defense markers such as Toll-like receptors TLR2 and TLR4 were also downregulated, suggesting chronic inflammation. These findings support a model of sustained dysregulated epithelial turnover due to impaired taste in LC-D. Saliva-based single-cell approaches offer promising tools for future diagnostics and mechanistic studies of taste systems.

## 1. Introduction

Gustation, the sense of taste, is mediated by taste buds located chiefly on the tongue but also across the palate, pharynx, and epiglottis. Dysfunction of taste can arise from several causes such as genetic variants in taste receptor genes and impaired taste cell regeneration due to infection or inflammation [1,2,3]. Despite their multifactorial etiology, taste disturbances are frequently overlooked in clinical practice [4]. The COVID-19 pandemic, caused by severe acute respiratory syndrome coronavirus 2 (SARS-CoV-2), heightened the awareness of chemosensory impairments, as taste and smell disturbance emerged as hallmark symptoms of the infection [5]. More concerning is the persistence of taste dysfunction for months to years, particularly impaired bitter taste, constituting a predominant symptom of long COVID (LC) even among individuals who were asymptomatic or mildly symptomatic during initial infection [6,7,8,9,10]. Impaired taste sensitivity could increase the risk for other co-morbid conditions in LC such as chronic fatigue and new-onset diabetes [11,12].

Assessment of taste sensitivity traditionally relies on subjective questionnaires or psychophysical tests using defined tastant concentrations [4,6]. Few studies have investigated taste dysfunction at the molecular level, although physiological responses of taste buds to tastants are well characterized [13,14,15]. Each taste bud contains two main types of epithelial cells: specialized taste receptor cells (TRCs) that respond to specific tastants, and supporting cells, which help maintain the structure and function of the bud [16,17]. The integrity of this sensory system depends on the dynamic cellular turnover, with the average lifespans ranging from 2.5 to 3 days for epithelial cells and from 8 to 10 days for TRCs [18,19]. This continual renewal, differentiation, maturation, and exfoliation is tightly regulated to maintain oral homeostasis and taste sensitivity [3,20]. One of the proposed mechanisms for taste dysfunction is the increased apoptosis and extrusion of taste cells [2,3].

Modern advances in single-cell RNA sequencing (scRNA-seq) have revolutionized our understanding of cellular heterogeneity across tissues and biofluids [21,22,23]. A recent scRNA-seq analysis of nasal wash samples from COVID-19 patients revealed reduced interferon signaling and antigen presentation in epithelial cells following SARS-CoV-2 infection [24]. Since epithelial cells were shown to be infected by SARS-CoV-2 and human saliva contains approximately 4 × 10^5^ epithelial cells/mL, with varying percentages of viable cells that function as cargo for biological or pathogenic molecules [25,26,27], it follows that studying the salivary epithelial cells (SECs) may reveal molecular mechanisms of taste loss. In this study, our primary objective was to characterize the single-cell transcriptomic landscape of SECs in LC individuals with bitter taste dysfunction (LC-D) compared to healthy controls.

**Figure 1:**
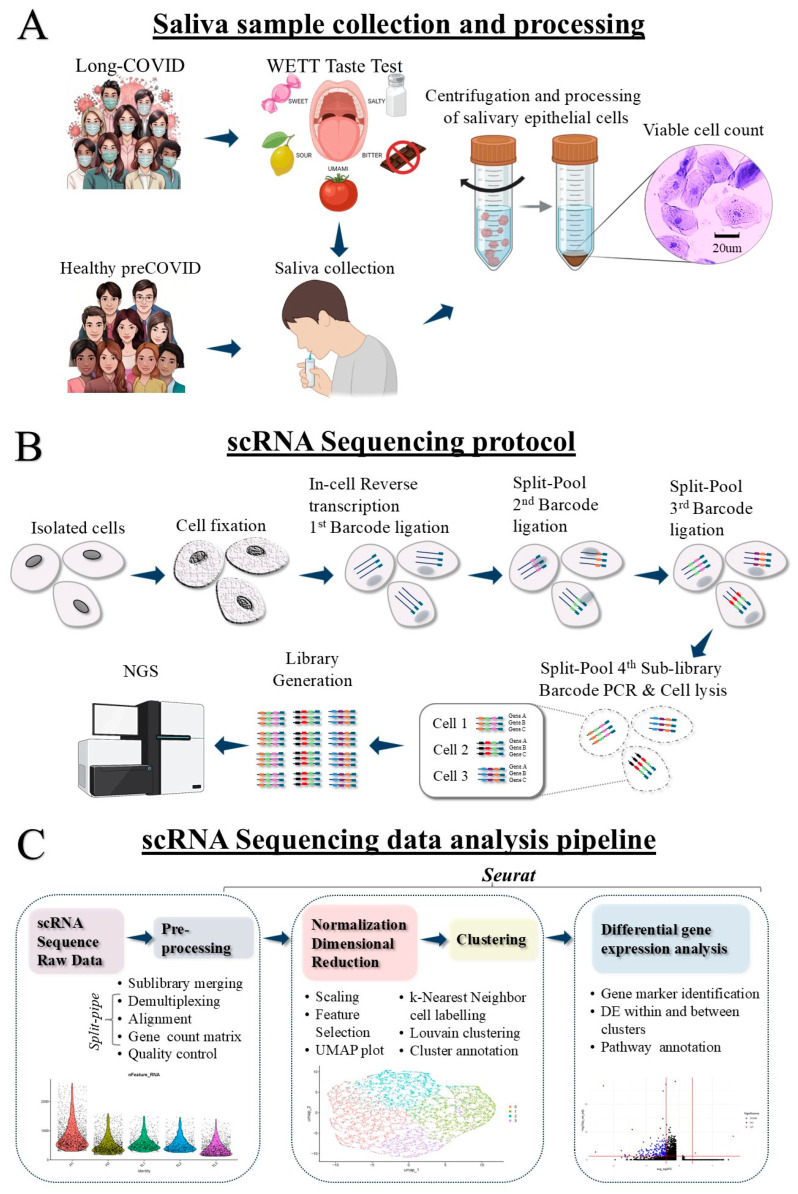
A schematic overview of the single-cell RNA sequencing (scRNA-seq) workflow for profiling salivary epithelial cells (SECs). (**A**) Viable epithelial cells isolated from unstimulated whole saliva of healthy and LC individuals with persistent dysgeusia (LC-D) were prepared for scRNA analysis. (**B**) scRNA-seq was performed using the Parse Biosciences Evercode Whole Transcriptome kit. Fixed SECs were processed through a series of in-cell reverse transcription and four rounds of split-pool barcoding. (**C**) Datasets resulting from sequencing were analyzed using the Seurat pipeline.

Our unbiased single-cell transcriptomic analysis revealed distinct clusters of epithelial cells at varying states of differentiation in LC-D, marked by a global downregulation of genes linked to epithelial homeostasis and synaptic signaling (Figure 1). More pertinently, there was a downregulation of multiple Type II TRC genes known to play a critical role in bitter taste perception. This is the first scRNA-seq investigation of SECs uncovering novel transcriptomic signatures of taste dysfunction. Our findings support SECs as a robust, non-invasive biospecimen with high potential for repeated, longitudinal sampling in clinical and translational research.

## 2. Results

### 2.1. High Prevalence of Bitter Taste Impairment in Long COVID (LC)

**Figure 2:**
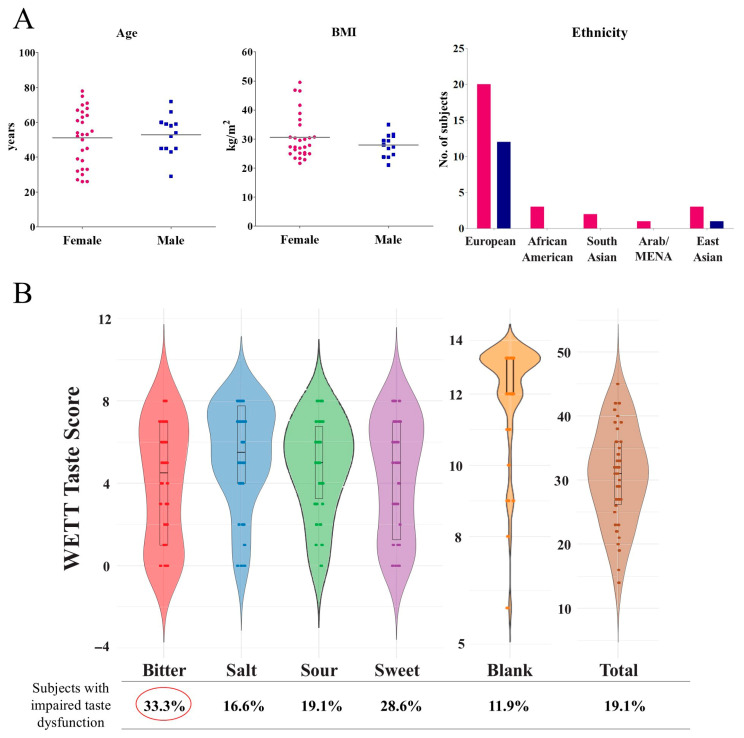
Demographic characteristics, taste scores, and SEC viability in LC. (**A**) A summary of age and BMI distribution among female (pink) and male (blue) participants in the LC cohort and the ethnic distribution of the study cohort, with most participants self-identifying as being of White/European ancestry. (**B**) Violin plots depicting individual and group-level taste scores across four basic tastants (sour, sweet, salty, bitter) and blank (phantom taste) responses, as well as total taste score distributions. The percentage of participants exhibiting impairment for each tastant is shown below the *x*-axis.

The average age of our LC cohort was 51.7 ± 14 years, the male-to-female ratio was 13:28 and the average BMI was 29.7 ± 6.7 (Figure 2A). Objective assessments of taste in LC subjects showed that 30% of individuals (13/42) exhibited impaired bitter taste, followed by 29% (12/42), 17% (7/42) and 19% (8/42) exhibiting impaired sweet, sour and salty perception respectively [28]. Complete ageusia was observed in 5%, and phantom or faulty taste (perceived in response to blank strips) was observed in 11.9% of individuals in the LC cohort. Interestingly, individuals with low total taste scores largely did not report phantom tastes, with one exception (Figure 2B).

### 2.2. The scRNA-seq of SECs from LC-D and Healthy Controls

SECs were screened for viability prior to their fixing for scRNA sequencing (Figure S2). SECs from six LC individuals with impaired bitter taste scores (≤1) (LC-D) were selected for scRNA sequencing. Six cryopreserved SECs derived from pre-COVID saliva were included as healthy controls [21]. The samples processed for scRNA-seq contained a total of 8472 cells (3756 LC-D and 4716 healthy) with a median of 585 genes per cell. Quality control filtered out cells with a gene count >5 times the mean absolute deviation (MAD), with a mitochondrial content >10%, or designated as doublets by scDblFinder [29]. This resulted in a post-QC set of 7321 cells (3293 LC-D and 4028 healthy).

**Figure 3:**
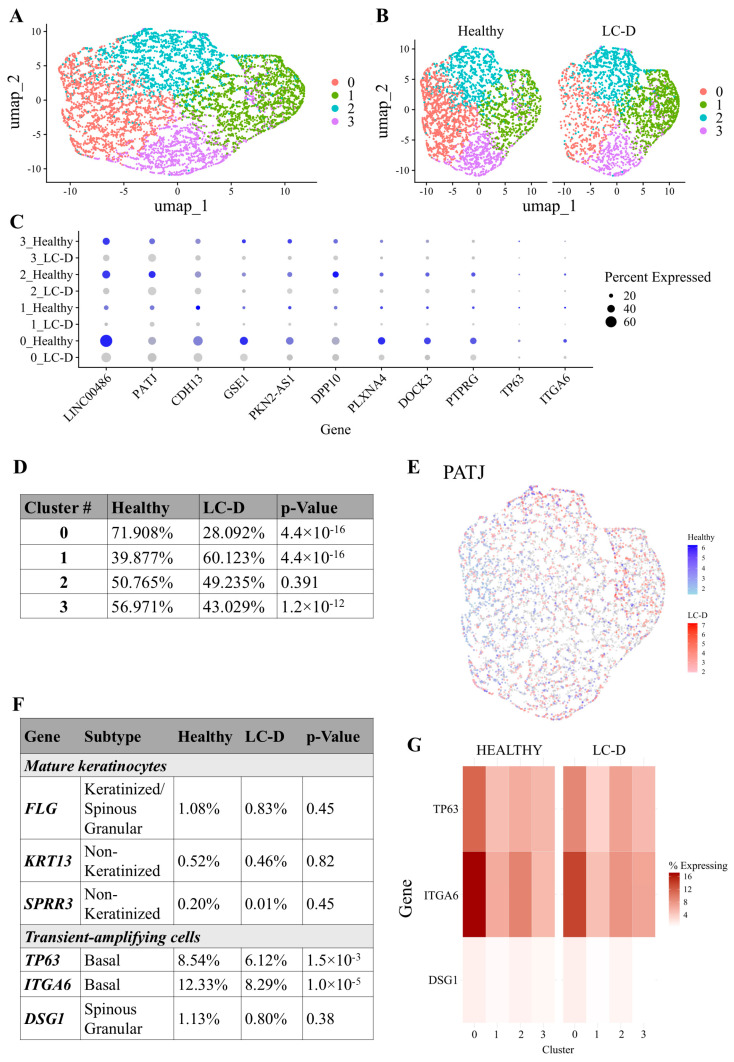
Cluster analysis of SECs. (**A**) UMAP visualization of 7321 SECs grouped into four transcriptional clusters (clusters 0–3) using Seurat-based clustering. (**B**) UMAP plot colored by sample type shows differential cluster occupancy by healthy and LC-D cells. (**C**) Dot plot showing expression of select markers across healthy (blue) and LC-D (grey) clusters. Dot size represents proportion of cells expressing each gene, and color intensity reflects average expression. (**D**,**F**) Table showing percentage of (**D**) cells in each cluster and (**F**) epithelial cell subtype markers in the indicated group with Benjamini–Hochberg (BH)-corrected *p*-values. (**E**) UMAP plot of PATJ confirming epithelial identity. (**G**) Heatmap of select marker genes. SECs: salivary epithelial cells; LC: long COVID; LC-D: LC with low bitter score (≤1); PATJ: human PALS1-associated tight junction protein.

Normalization, scaling, dimensionality reduction and clustering using the Seurat pipeline identified four transcriptionally distinct cell clusters (Figure 3A,B). Further analysis showed that the clusters 0 and 3 contained significant enrichment of cells from the healthy group (72% and 57% respectively) as compared to the LC-D cohort (28% and 43% respectively) (*p* = 4.4 × 10^−16^ and *p* = 1.2 × 10^−13^ respectively). Cluster 1 cells showed a marked predominance of LC-D (60%) over the healthy cohort (40%) (*p* = 4.4 × 10^−16^). Cluster 2 did not exhibit any significant difference in cell distribution between the two cohorts (Figure 3C,D).

Cell type annotation using Seurat revealed that the epithelial differentiation marker PALS1-associated tight junction protein (PATJ) was ubiquitously expressed across all clusters [30] (Figure 3C,E). Similarly, markers of epithelial structural integrity, including filaggrin (FLG), keratin 13 (KRT13), and small proline-rich protein 3 (SPRR3) [31], remained relatively stable between healthy and LC-D samples, indicating a consistent presence of mature epithelial cells in both cohorts (Figure 3F,G). In contrast, the expression of Desmoglein 1 (DSG1), a critical cell–cell adhesion molecule in suprabasal squamous epithelia, showed a downward trend in the LC-D cohort, suggesting perturbation in terminal differentiation (Figure 3F,G). Further, TP63, a master regulator of epithelial development and basal cell proliferative capacity, was significantly reduced in the LC-D samples (Figure 3F,G). A similar decrease was detected in integrin alpha-6 (ITGA6), a critical mediator of cell adhesion and epithelial stem cell maintenance. Collectively, these transcriptional changes indicate a disruption in the population of transit-amplifying cells required for epithelial renewal in the LC-D cohort (Figure 3F,G).

### 2.3. Type II and Type III Taste Receptor Cells Are Reduced in LC-D

**Figure 4:**
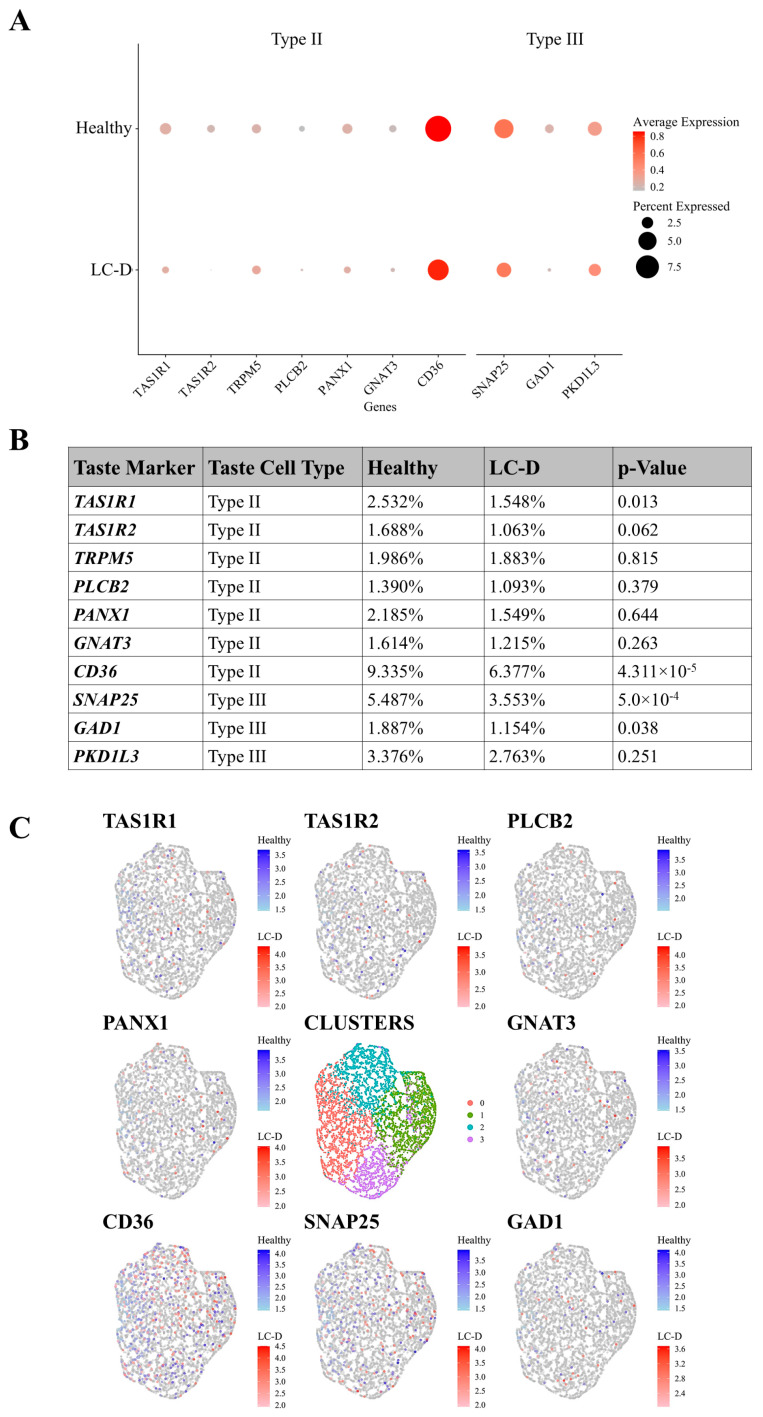
Analysis of taste cell marker expression. (**A**) Dot plot showing average expression (color intensity) and percent expression (dot size) of taste cell markers in healthy and LC-D cohorts, categorized by taste cell type (Type II, III). (**B**) Table showing percent expression of markers and BH-corrected *p*-values. (**C**) UMAP plots of taste cell subtype markers. Shown in center is reference cell cluster. SECs: salivary epithelial cells; LC: long COVID; LC-D: LC with low bitter score (≤1).

To explore TRC-specific genes in our dataset, we queried for known TRC markers curated from publicly available datasets [31,32,33] (Figure 4A). TRCs are classified as Type I supporting cells; Type II cells that detect sweet, bitter and umami tastes; and Type III cells for sour perception. Type IV are basal progenitor cells that regenerate TRCs every 7–10 days [16,33]. The percentage of cells expressing any type of taste cell marker in the LC-D cohort ranged between 1.1% and 6.4%; this was lower than that in the healthy cohort, which ranged between 1.4% and 9.3% (Figure 4A–C). Our data predominantly showed differences in Type II and Type III TRCs [2]. Of the seven Type II taste cell markers annotated, the percentages of cells expressing TAS1R1, TAS1R2, and CD36 were significantly lower in the LC-D cohort (1.54%, 1.06% and 6.38% respectively) compared to those in the healthy group (2.53%, 1.69% and 9.34% respectively). These findings agree with the low bitter scores recorded by individuals in the LC-D cohort. Among the Type III taste cell markers, synaptosome-associated protein 25 (SNAP25) and glutamate decarboxylase 1 (GAD1) were observed in a lower percentage of cells in the LC-D (3.55% and 1.15% respectively) group compared to the healthy cohort (5.49% and 1.89% respectively).

### 2.4. Validation of scRNA seq Data of SECs

**Figure 5:**
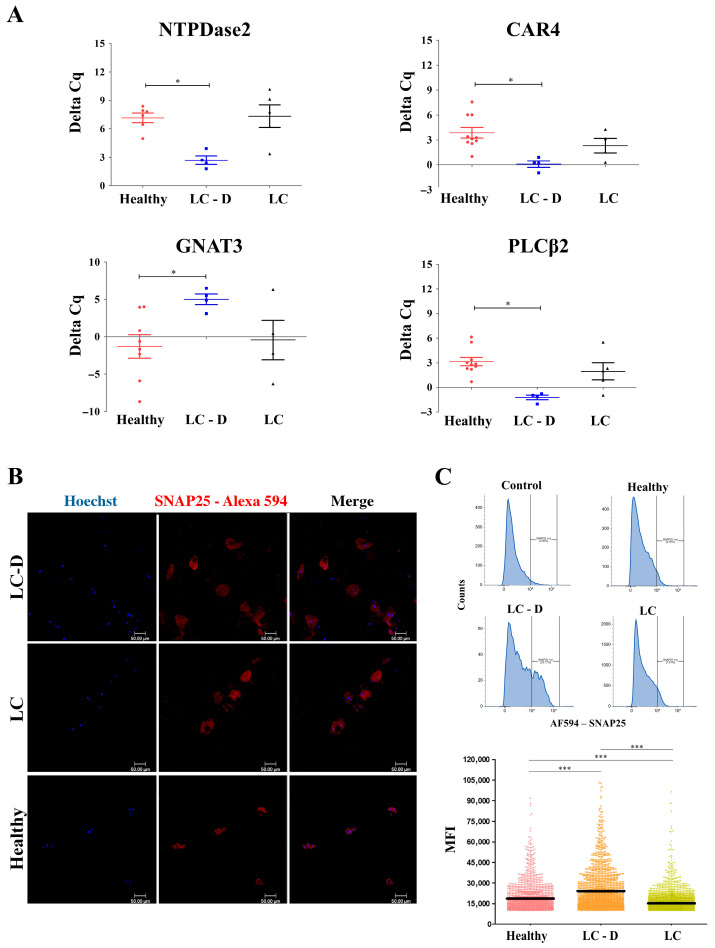
Quantitative PCR and immunofluorescence of TRC markers: (**A**) ΔCq for NTPDase2 (Type I), CAR4 (Type III), PLCB2 and GNAT3 (Type II) across the indicated groups; * = *p* < 0.05. (**B**) Immunofluorescence staining for the Type III TRC SNAP25 (red, Alexa 594) in SECs in indicated groups. Nuclei were counterstained with Hoechst (blue). (**C**) Flow cytometry analysis shows a histogram (top panel) quantification of SNAP25 expression (bottom panel) ), *** = *p* < 0.0001. TRC: taste cell receptor; LC-D: LC with low bitter score (≤1); LC: long COVID without dysgeusia.

To validate the scRNA data we quantitated the expression of select taste cell markers including a Type I TRC marker, nucleoside triphosphate diphosphohydrolase 2 (NTPDase2); two Type II TRC markers, PLCβ2 (phospholipase C Beta2) and GNAT3 or α-gustducin; and a Type III marker, CAR4, by RT-PCR [2,34]. The Kruskal–Wallis test suggested significant differences between the LC, LC-D, and healthy groups in the expressions of NTPDase2 (H = 7.5, *p* = 0.023), PLCβ2 (H = 9.8, *p* = 0.007), CAR4 (H = 9.85, *p* = 0.011) and GNAT3 (H = 5.4, *p* = 0.06). The pair-wise Mann–Whitney test showed that the expressions of NTPDase2, PLCβ2 and CAR4 were significantly higher in SECs from the LC-D cohort (ΔCq = 2.7 ± 0.5, ΔCq = −1.2 ± 0.3, ΔCq = 0.08 ± 0.4 respectively) than in SECs from healthy controls (ΔCq = 7.2 ± 0.5, ΔCq = 3.2 ± 0.5, ΔCq = 3.9 ± 0.6 respectively) (Figure 5A) [2,35]. The expression of GNAT3 was significantly reduced in SECs from the LC-D cohort (ΔCq = 5 ± 0.7) compared to those from healthy controls (ΔCq = −1.3 ± 1.6) (Figure 5A). Allelic variations in GNAT3 have been associated with the threshold and preferred intensity of sweet taste perception [36].

We next assessed the protein expression of select taste cell markers by immunofluorescence [6]. A Type III TRC marker, SNAP25, exhibited increased expression in SECs from the LC-D samples as compared to cells from LC without taste dysfunction and the healthy controls (Figure 5B). Flow cytometry analysis also showed that the mean fluorescence intensity (MFI) of SNAP25 staining was significantly elevated in the LC-D cohort as compared to the LC cohort without taste dysfunction, and that in the LC without taste dysfunction cohort was elevated as compared to that in the healthy group (Figure 5C). The discrepancy in the transcript and protein expression of SNAP25 could be attributed to the differential efficiency in translation of mRNA across cell types or technical differences such as assessment at the single-cell (scRNA seq) versus the multiple-cell (IF/flow cytometry) level.

### 2.5. Pathways Associated with Taste Are Downregulated in LC-D

**Figure 6:**
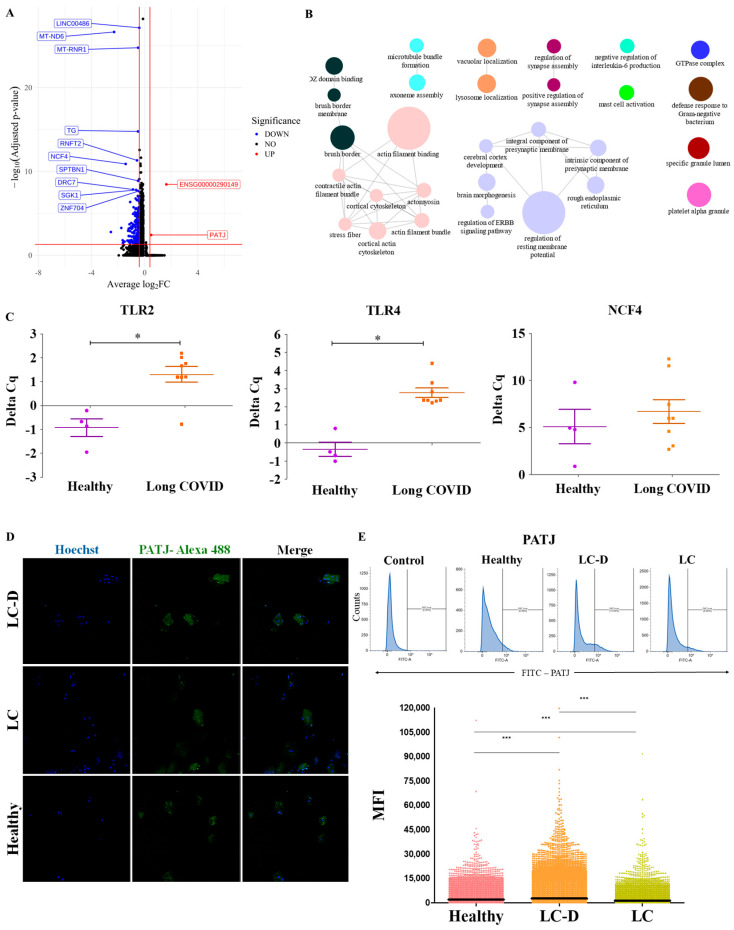
Differentially expressed genes (DEGs) in SECs. (**A**) A volcano plot showing DEGs in SECs from LC-D and healthy controls; labels indicate significantly upregulated genes (red) and downregulated genes (blue). False discovery rate thresholds are shown as red lines. (**B**) A network diagram of the enriched Gene Ontology (GO) terms found in the downregulated DEGs. The size of the nodes is −log_10_ (*p* value). Ontology term groups are differentiated by color. (**C**) RT-PCR of TLR-2, TLR-4 and NCF4 expression in SECs. * = *p* < 0.05. (**D**) Representative immunofluorescence showing PATJ expression in SECs in indicated groups. Nuclei were counterstained with Hoechst. (**E**) Flow cytometric analysis showing a histogram and the mean fluorescence intensity (MFI) of PATJ expression across groups. *** = *p* < 0.0001. SECs: salivary epithelial cells; LC: long COVID; LC-D: LC with low bitter score (≤1).

Global transcriptomic changes assessed by differential gene expression analysis revealed dominant downregulation of 171 genes in the LC-D cohort, suggesting functional impairment or degeneration in the affected cells (Figure 6A). Interestingly, PATJ, the only gene observed to be significantly upregulated in the LC-D cohort, is a tight-junction regulating gene shown to interact with SARS-CoV-2, supporting its increase in the LC-D cohort [30,37,38].

Gene Ontology enrichment analysis for the 171 downregulated genes identified a total of 16 groups from 39 individual GO terms, with 12 groups being significant after Bonferroni step-down as performed by ClueGo [39] (Figure 6B). Among the significantly enriched categories were genes associated with actin filament-related pathways, consistent with the observed downregulation of DSG1, TP63 and ITGA6 in the LC-D cohort (Figure 3F,G). Pertinent to taste dysfunction, pathways related to synapse assembly and function, “brush border” and “microvillus organization” genes, and components of the GTPase complex, critical for G-protein-coupled receptor (GPCR) signaling, were downregulated in the LC-D cohort [1,3]. In addition, multiple immune-related pathways including “mast cell activation,” “platelet alpha granule,” and “defense response to Gram-negative bacterium” and the interleukin-6 (IL-6) signaling pathway were also downregulated (Figure 6B).

To validate the predicted gene pathways, we assessed the expressions of select immune-related genes by RT-PCR. Previously SECs have been shown to express TLR2 and TLR4, bind pathogen-associated molecular patterns, and elicit immune responses [22]. TLR2 and TLR4 have been suggested as potential receptors for SARS-CoV-2, and their downregulation could be the result of a mechanism adopted by the virus to avoid detection by innate immune cells [40,41]. We observed that the expression of TLR2 and TLR4 (Figure 6C) was significantly reduced in LC SECs (ΔCq: 1.3 ± 0.33 and 2.8 ± 0.27 respectively) compared to healthy controls (ΔCq: −0.92 ± 0.36 and −0.35 ± 0.39 respectively). Further, the expression of neutrophil cytosolic factor 4 (NCF4), a gene that encodes the p40-phox subunit of the NADPH oxidase enzyme complex and functions as a bactericidal agent [42], was also decreased in the LC cohort (ΔCq: 6.7 ± 1.5) as compared to healthy controls (ΔCq: 5.1 ± 1.8), although the difference was not statistically significant (Figure 6C).

We next assessed the expression of PATJ, the epithelial structural marker shown to be upregulated in the DEG analysis (Figure 6A), by immunofluorescence. We observed that PATJ exhibited stronger expression in the cytoplasm of epithelial cells in the LC-D cohort as compared to that in the LC group without taste dysfunction and healthy controls (Figure 6D). This finding was corroborated by flow cytometry, with the signal intensity of PATJ being significantly higher in SECs from the LC-D cohort (average MFI: 2564 ± 15.8) than in those from the LC (average MFI: 1235 ± 7.7) or healthy control cohorts (average MFI: 1970 ± 13.4) (Figure 6E). These observations strongly support the upregulation of PATJ in alignment with the DEG observed in scRNA-seq.

## 3. Discussion

The COVID-19 pandemic provided a unique opportunity to study taste perception, particularly since 5–30% of individuals continue to experience persistent taste deficits post-SARS-CoV-2 infection [7,9,10,11]. Perturbation in the homeostasis between the loss and replenishment of TRCs is implicated in driving taste deficits [3,43]. We report the first scRNA-seq profiling of human SECs and their relevance in investigating disease pathology. The Evercode™ technology used in this study avoids microfluidics, thereby overcoming limitations imposed by large cell sizes. The technique enabled high-throughput scRNA-seq of large, heterogeneous SECs via a combinatorial barcoding method [34]. Collectively, our observations suggest novel transcriptomic signatures of taste dysfunction.

The downregulation of genes involved in actin filament and intercellular communication pathways in the LC-D cohort suggests perturbations in cytoskeletal remodeling, broad structural alterations in transient-amplifying cells, and impaired epithelial maintenance with consequent effects on taste perception. Consistent with this, we also observed reduced expression of keratin 14, a stem cell marker associated with taste progenitor cells, in LC-D SECs (3,6). Our observations align with prior reports of decreased levels of stem cell markers, SOX2, SHH, and Ki67, in fungiform papillae of individuals with taste disorders [2,44], supporting the hypothesis that taste cell lineage maintenance is compromised in LC-D. Interestingly, PATJ, a tight-junction regulating gene that was significantly upregulated in the LC-D cohort, has been shown to interact with the SARS-CoV-2 envelope protein [30,37,38]. These observations suggest sustained dysregulation of epithelial homeostasis following SARS-CoV-2 infection.

Importantly, pathways related to synapse assembly and function were downregulated in the LC-D cohort, which is particularly relevant given the essential role of synaptic communication in transmitting taste signals from sensory cells to afferent neurons [3]. Downregulation of “brush border” and “microvillus organization” genes could reflect impaired formation of microvilli on taste cells, structures essential for tastant detection. Components of the GTPase complex, critical for G-protein-coupled receptor (GPCR) signaling involved in sweet, umami, and bitter taste detection, were also significantly downregulated [1,3].

One of the key findings from our study is the reduced expression of Type II TRCs in the LC-D cohort. Type-II TRCs make up about 25% of taste bud cells and are crucial for detecting bitter and umami flavors [1,2,3]. Specifically, the downregulation of the TAS1R1, TAS1R2, and CD36 Type II TRC-associated genes aligns with the decreased bitter taste sensitivity observed in our LC cohort. Further, elevated expression of NTPDase2, SNAP25 and CAR4, proteins that facilitate the release of neurotransmitters from Type II and Type III TRCs, in SEC samples from LC individuals with dysgeusia suggests increased extrusion of taste cells contributing to the decreased bitter taste perception. We also observed that the expression of semaphorin 3A, a guidance molecule for neuronal specificity in bitter taste transmission, was significantly reduced in the LC-D group (data not shown) [43]. Together, the reduced expression of TRC genes and of genes involved in cytoskeletal integrity and synaptic transmission may interfere with microvillus formation and neurotransmitter release, further affecting taste perception. However, given substantial genetic variability in TRCs (including polymorphisms in TAS2R38), along with known effects of ethnicity, sex, and aging on taste perception and receptor expression, the variability observed likely reflects both disease-associated transcriptional changes and demographic heterogeneity, warranting validation in larger and more diverse cohorts [45,46].

Inflammation-mediated epithelial damage is suggested to be an important mechanism leading to taste dysfunction. Emerging evidence suggests the oral mucosa as a persistent reservoir for SARS-CoV-2 and TLR2/4 as its potential host receptors [47]. We observed decreased expression of bacterial defense markers, TLR2 and TLR4 and downregulation of genes involved in the IL-6 signaling pathway, consistent with disturbed apoptosis with potential effects on TRC regeneration [17,48]. Collectively, these findings suggest that concurrent impairments in taste receptor structure, signal transduction, and local immune responses contribute to the persistent dysgeusia observed in LC-D.

## 4. Materials and Methods

### 4.1. Study Participants

An electronic survey was sent to individuals aged 18 years and older who had previously agreed to be notified about COVID-19 studies registered at IU School of Medicine’s COVID-19 Research Registry [49]. Definition of post-COVID: Individuals with a history of a SARS-CoV-2-positive laboratory (RT-PCR) test and self-reported taste loss following the positive test lasting for longer than 60 days were recruited [7,49]. This included 42 individuals.

### 4.2. Taste Test

To objectively measure taste perception all participants completed the objective chemosensory evaluation using the Waterless Empirical Taste Test ™ (WETT^®^) (Sensonics International, Haddon Heights, NJ, USA) [28]. The test items consist of 53 paper taste strips coated on one end with increasing concentrations of dried sucrose (0.20, 0.10, 0.05, or 0.025 g/mL), citric acid (0.025, 0.05, 0.10, or 0.20 g/mL), sodium chloride (NaCl; 0.0313, 0.0625, 0.125, or 0.25 g/mL), caffeine (0.011, 0.022, 0.044, or 0.088 g/mL), monosodium glutamate (MSG; 0.017, 0.034, 0.068, or 0.135 g/mL), or no stimulus [28]. The WETT presents each stimulus from weak to strong concentration in an ascending sequence in the first half and in reverse presentation, going from strong to weak concentrations, in the second half. Different tastants are presented in a random order except that no tastant immediately follows itself. Blanks are interspersed after the higher concentrations of caffeine, NaCl, and citric acid to remove the residual stimulus. The scores for each of the five tastes range from 0 (low) to 8 (strong) perception. The WETT is highly reliable, with test–retest and split-half reliability coefficients of 0.92 and 0.88, respectively [28]. Subjects with WETT scores < 2 were considered dysgeusic.

### 4.3. Specimen Collection and Processing

Unstimulated whole saliva (UWS) was collected prior to taste-testing from all long-COVID participants by the passive drooling method as described in [6] (Figure 1A). All samples were processed within 30 min of collection and centrifuged at 3200 rpm for 10 min at 4 °C to separate clarified from cellular saliva. Red blood cells were removed by ZAP-OGLOBIN II Lytic Reagent (Beckman Coulter, Fullerton, CA, USA). After passing through a 40µ filter, the fraction enriched with SECs was stored at −80 °C in cell freezing media until further analysis. Cryopreserved SECs from pre-COVID saliva samples were obtained from the saliva bank and constituted the healthy controls [24].

### 4.4. scRNA Library Construction and Sequencing

To accommodate for the large size and heterogeneity of SECs, we used Parse Biosciences’ Evercode™ Whole Transcriptome technology that employs microfluidics for combinatorial barcoding of heterogenous SECs [50] (Figure 1B). Twelve SEC samples (six each from healthy individuals and LC-D) were prepared by dissociating SECs and filtering them through a 70 μm strainer to ensure a uniform suspension. The Evercode^TM^ requires a minimum of 80% viability for further processing. The viability of the SECs was determined by counting in a hemocytometer (BD PharMingen, San Jose, CA, USA) cells stained with acridine orange and propidium iodide (AOPI) (AAT Bioquest, Pleasanton, CA, USA). In our samples, the viability of SECs ranged between 80% and 95% (Figure S2). A minimum of 75,000 cells per sample were utilized to optimize cell recovery and ensure robust transcriptomic representation. SECs from each saliva sample were fixed using Parse Evercode Cell Fixation v3 (Parse Biosciences, Seattle, WA, USA). The combination of barcodes enables the unique mapping of reads to respective samples and individual cells. Libraries were prepared using the Parse Evercode Whole Transcriptome kit (V3) and sequenced on the Illumina NovaSeq by using the services of the Northwestern University Sequencing Core (NuSeq), Chicago, IL, USA.

### 4.5. scRNA-seq Data Analysis

Sequencing of the twelve samples was performed, generating CBCL files for eight sub-libraries (Figure 1C). These files were converted into FASTQ format using Illumina’s BCL Convert software v4.3.6 [51]. The FASTQ files were processed through Parse Biosciences’ split-pipe pipeline, resulting in the creation of count matrices for each sample [49]. Quality control and analysis were conducted using the Seurat package [52]. Cells that had either a gene or molecule count of greater than 5 mean absolute deviations (MADs) were filtered out, as well as cells with a mitochondrial content greater than 10%. Doublet scores were assigned with scDblFinder 1.12.0 [29], and barcodes determined to be doublets were removed [53]. The samples were merged, and the gene counts were normalized using log normalization with a scale factor of 10,000. Variable features were identified through the variance-stabilizing transformation method. The data were scaled, subjected to dimensionality reduction using Principal Component Analysis (PCA) and visualized with the Uniform Manifold Approximation and Projection (UMAP) technique. Clustering was achieved using the K-Nearest Neighbors (KNN) algorithm to identify distinct cell populations. Differentially expressed genes (DEGs) between LC-D and healthy cells were calculated with a Wilcoxon Rank Sum test. The DEGs were input to ClueGO to calculate a network of enriched Gene Ontology terms [39].

### 4.6. Immunocytochemistry and Flow Cytometric Analysis

SECs were fixed with 4% paraformaldehyde in PBS on a coverslip for 20 min at room temperature, followed by three washes with PBS. The cells were then permeabilized with 0.1% Triton X-100 in PBS for 10 min followed by blocking with 5% BSA in PBS for 1 h at room temperature to minimize nonspecific binding. The primary antibodies used included anti-human PALS1-associated tight junction protein (PATJ) (Clone 3C6A4, Proteintech, Rosemont, IL, USA) and anti-human synaptosome-associated protein 25 (SNAP25) (Clone SP12, Santa Cruz Biotechnology, Santa Cruz CA, USA). The cells were incubated overnight at 4 °C with primary antibodies at 1:250 IN 1% BSA in PBS. After washing with PBS the cells were exposed to secondary antibodies conjugated to fluorescent dye for 1 h at room temperature. For nuclear counterstaining, the cells were incubated with Hoechst 33,342 (1 µg/mL) for 10 min. Following the final washes with PBS, the coverslips were mounted onto slides, and immunofluorescent images were acquired using a confocal microscope (Stellaris 5, Leica, Wetzlar, Germany). Samples without primary antibody samples were used as negative controls [15,54]. For flow cytometric analysis, prior to staining as described above, cells were resuspended in 200 µL PBS and filtered through a 70 µm cell strainer to ensure a single-cell suspension. Flow cytometric analysis was performed using a SORP X20 flow cytometer (BD Biosciences, San Jose, CA, USA) [55,56]. Collected data were analyzed using Floreada (https://floreada.io, Accessed date: 27 March 2025). Compensation was applied where necessary, and gating strategies were set using appropriate negative controls (unstained and single-stained controls).

### 4.7. Real-Time qPCR (RT-qPCR)

Total RNA was extracted from SECs using the mirVana™ miRNA Isolation Kit (ThermoFisher Scientific, Waltham, MA, USA) according to the manufacturer’s instructions. cDNA was synthesized from 200 ng of RNA using the RevertAid First Strand cDNA Synthesis Kit (ThermoFisher) using Random Hexamers as primers. RT-qPCR was performed on a CFX96 (BioRad, Hercules, CA, USA), using PowerUp SYBR Green Master Mix (Applied biosystems, Foster City, CA, USA) and 0.625 μM of gene-specific primers (Integrated DNA Technologies Coralville, IA, USA) as a 20 µL reaction using the manufacturer’s protocol [26]. At the end of each RT-qPCR run, a melting curve analysis was conducted to detect any nonspecific PCR products. Amplification of small proline-rich 2A (SPRR2A), abundantly expressed in oral epithelial cells, was performed to provide a reference gene for SECs [26]. All samples were run in duplicate. The primers and the PCR protocols are provided in Supplementary Table S1, Figure S1 in accordance with MIQE guidelines. ΔCq values (Cq target–Cq_SPRR2A), which represent normalization of the target gene to the internal reference gene, were calculated. Data are presented as the mean ΔCq ± standard deviation (SD).

### 4.8. Statistical Analysis

Statistical analysis for the scRNA dataset was performed using R. The Chi-Square tests for independence within the stats package were used to test for associations between healthy controls and LC-D. The *p*-values were adjusted for multiple hypotheses by Benjamini–Hochberg (BH) correction. Comparative analyses of ΔCq values were performed by parametric and non-parametric tests, and the data presented are results from a Kruskal–Wallis test, confirmed by a pair-wise Mann–Whitney test. Statistical significance in flow cytometric values was assessed by a two-tailed t-test using GraphPad Prism 5 (GraphPad Software, LLC, San Diego, CA), and results are presented as the mean ± standard deviation (SD). *p*  <  0.05 was considered statistically significant.

### 4.9. Figure Generation

Figures was formatted on Microsoft Powerpoint. scRNA sequemcing figures figures were compiled into panels using Inkscape version 1.4.2.

## 5. Conclusions

Our single-cell transcriptomic analysis suggests that the bitter taste dysfunction in LC is marked by disrupted epithelial homeostasis, weakened synaptic signaling, and diminished Type II TRC activity. The reduced expression of genes involved in cell proliferation and differentiation supports impaired regenerative capacity in the taste epithelium, contributing to persistent sensory deficits in the LC-D group.

Nonetheless, our study has several limitations. A small sample size and cross-sectional design limit the generalizability of our data and prevent causal conclusions. We also did not measure viral load or monitor temporal changes. Challenges associated with Evercode^TM^ include sequencing partial transcripts and detecting fewer genes per cell, which contributed to inconsistent barcode incorporation and RNA loss in some samples. It is worth noting that while droplet and plate-based methods differ in their handling of cell size, plate-based approaches generally yield better RNA recovery for large or fragile cells [57,58].

Despite these limitations, our integrated approach of combining psychophysical, immunologic, and transcriptomic data offers a compelling framework for elucidating taste dysfunction in LC. This study represents the first scRNA-seq report on human SECs, establishing a foundation for saliva-based single-cell profiling as a scalable, non-invasive tool for diagnosing and tracking taste system disorders over time.

## Data Availability

The original contributions presented in this study are included in the article/Supplementary Material. Further inquiries can be directed to the corresponding author.

## Supplementary Materials

The following supporting information can be downloaded at https://www.mdpi.com/article/10.3390/ijms27072953/s1.
