# Supplementary material for: Single-Cell Transcriptomic Analysis of Salivary Epithelial Cells Reveals Large-Scale Dysregulation in Bitter Taste Dysfunction
Source: Int J Mol Sci. 2026 Mar 24;27(7):2953. doi: 10.3390/ijms27072953 (PMC13072927; doi:10.3390/ijms27072953)
Supplement: Supplementary file 1 [file ijms-27-02953-s001.zip › ijms-4129509-supplementary.pdf]

Supplementary information

Supplementary Table S1. qPCR primer sequences and amplification parameters

Gene	Primer	Sequence (5′–3′)	Amplicon Size (bp)	Slope	Efficiency (%)	Annealing Temp (°C)
NTPDase2	Forward	TCACACACGTCCATGTTTATCT	126	−3.58	90.25	54
	Reverse	AGAAGGGTTGTCTGCATAGC				
GNAT3	Forward	GTGAAAGCCATGACTACCCTTGG	123	−2.82	127.58	54
	Reverse	CTCAGCCAGTTGAGGTGTCATG				
PLCB2	Forward	GTCACCTGAAGGCATGGTCT	164	−3.21	104.89	54
	Reverse	AGGAGAGGCCTGAGAACTG				
CAR4	Forward	CATACCAGGCCAAACAGTTG	145	−3.07	111.71	54
	Reverse	GCCTCTTTCACATTCCTCG				
NCF4	Forward	AGAAGAGAGGCTTCACCAGCCA	121	-3.49	93.43	54
	Reverse	TCCTCCAGCTTGCTCTGCAAAG				

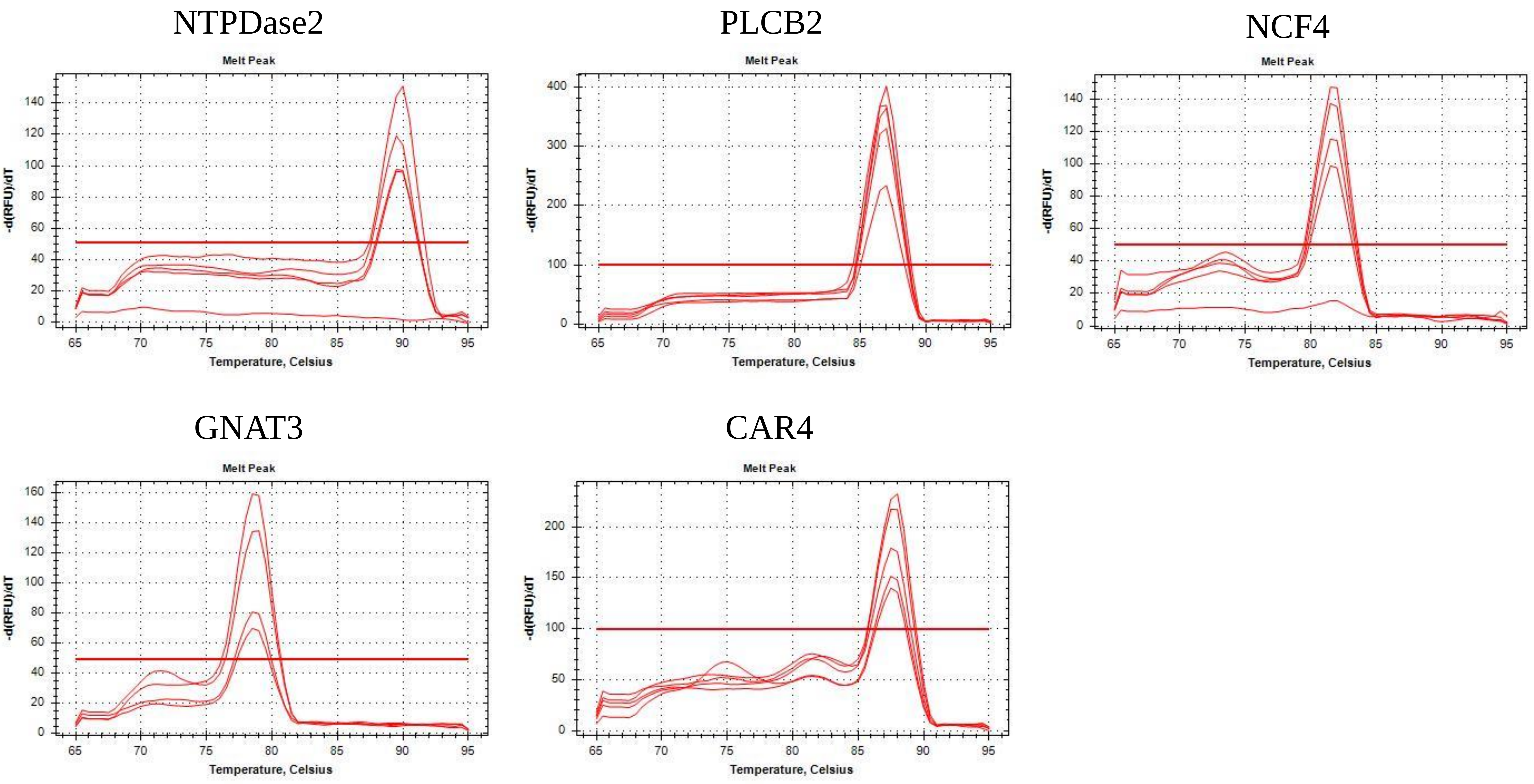

Supplementary Figure S1. Validation of qPCR Primers: Representative melt curve analysis of NTPDase2, PLCB2, GNAT3, CAR4 and NCF4 for testing primer specificity. Single peak indicating single product. Images were generated from CFX Maestro Software for CFX96 Real-Time PCR Instrument.

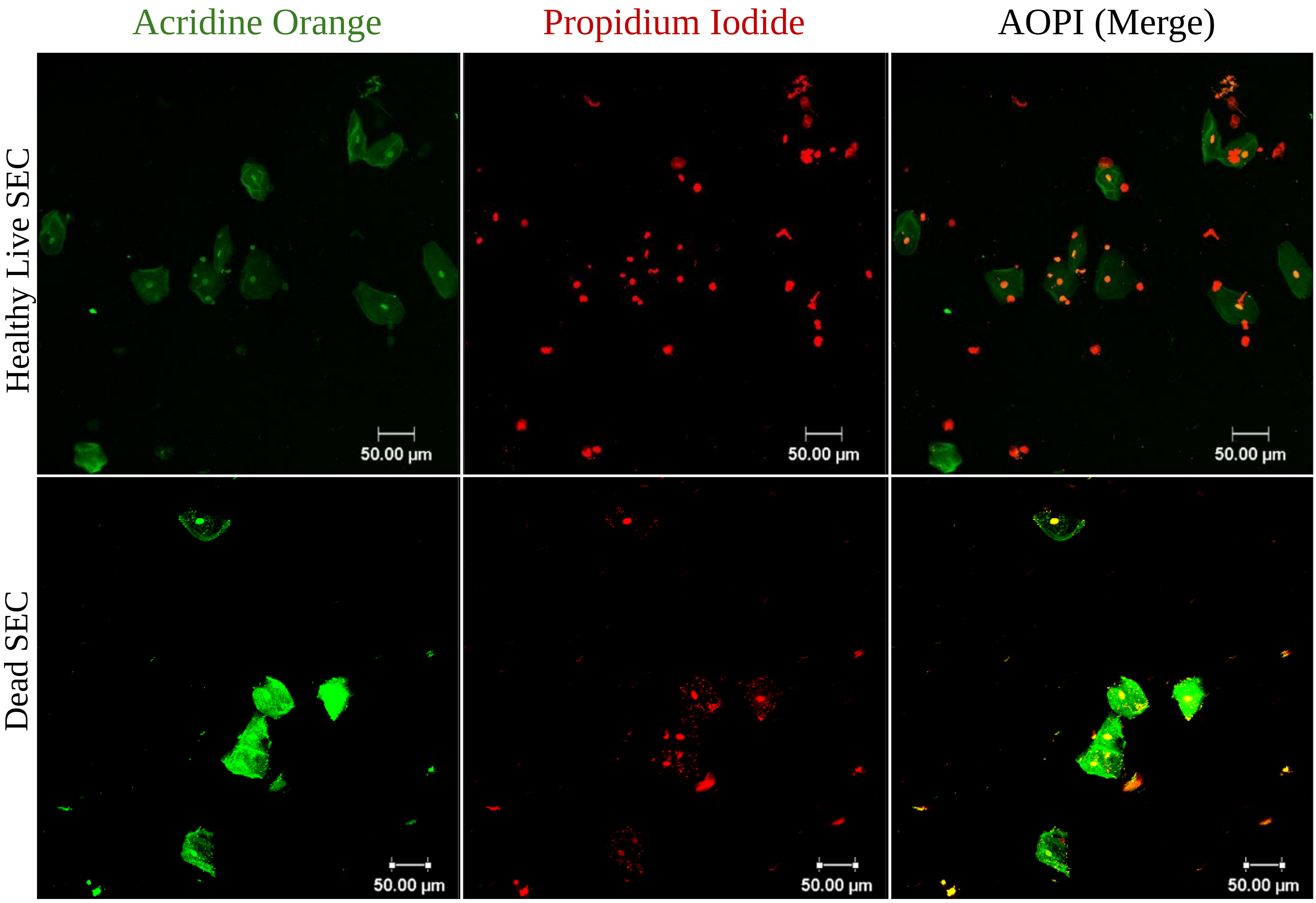

Supplementary Figure S2: Representative maximum intensity projection fluorescence images showing viability of SECs using acridine orange and propidium iodide. Merged AOPI (Acridine Orange/Propidium Iodide) images confirm the predominance of viable cells prior to single-cell RNA sequencing. Dead SEC which are brightly stained by AO are represented for comparison. SECs: Salivary epithelial cells, LC: Long-COVID, 20X, Leica Stellaris 5.
